# Melatonin as an Agent for Direct Pulp-Capping Treatment

**DOI:** 10.3390/ijerph17031043

**Published:** 2020-02-06

**Authors:** Julia Guerrero-Gironés, Antonia Alcaina-Lorente, Clara Ortiz-Ruiz, Eduardo Ortiz-Ruiz, María P. Pecci-Lloret, Francisco Javier Rodríguez-Lozano, Carlos M. Martínez, Antonio José Ortiz-Ruiz

**Affiliations:** 1 Special Care and Gerodontology Unit, IMIB-Arrixaca, Campus Regional de Excelencia Internacional “Campus Mare Nostrum”, University of Murcia, 30008 Murcia, Spain; juliaguerrero1@hotmail.com (J.G.-G.); mpilar.pecci@gmail.com (M.P.P.-L.); fcojavier@um.es (F.J.R.-L.); 2 Department of Integral Pediatric Dentistry, University of Murcia, 30008 Murcia, Spain; alcainalorentea@gmail.com; 3 Department of Physiology, School of Medicine and Biosanitary Research Murcian Institute (IMIB), University of Murcia, 30120 Murcia, Spain; clara@um.es; 4 Department of Histopathology, University Hospital Virgen de la Arrixaca, 30120 Murcia, Spain; edu_or@yahoo.es; 5 Experimental Pathology Unit, Biomedical Research Institute of Murcia-Arrixaca, IMIB-Arrixaca, 30120 Murcia, Spain; cmmarti@um.es

**Keywords:** direct pulp capping, melatonin, mineral trioxide aggregate, dentin bridge, hematoxylin–eosin, oxidative stress

## Abstract

Melatonin plays an essential role in the regulation of bone growth. The actions that melatonin exerts on odontoblasts may be similar to its action on osteoblasts. This research aimed to evaluate the pulp response to melatonin used for direct pulp capping to evaluate the antioxidant effect of melatonin administered orally and its influence on dental pulp. Direct pulp capping was performed on the upper molars of Sprague Dawley rats using melatonin or Mineral Trioxide Aggregate (MTA). The study groups were: MTA; Melatonin; MTA + Melatonin administered orally; and Melatonin + Melatonin administered orally. In the latter two groups, the animals drank water dosed with melatonin ad libitum (10 mg/100 mL). After 30 days, the animals were sacrificed, and 5 ml of blood, the kidneys, and the liver were extracted in order to evaluate oxidative stress using thiobarbituric acid reactive substances testing (TBARS). Fragments of the maxilla containing the study molars were prepared for histological evaluation. The degree of pulp inflammation and pulp necrosis, the presence of reparative dentin and dentin bridging the pulp chamber, the presence and regularity of the odontoblastic layer, and the presence of pulp fibrosis were evaluated. No significant differences were found between the four study groups for any of the studied histological variables. The oral administration of melatonin did not modify the local effects of MTA or melatonin on dental pulp, or reduce basal-level oxidative stress. The effect of melatonin on pulp is similar to that of MTA and may be used as an agent for direct pulp capping.

## 1. Introduction

Mineral Trioxide Aggregate (MTA) is commonly used in vital-pulp therapy, which has had histological, radiological, and clinical success. Vital-pulp therapy includes procedures such as direct pulp capping, indirect pulp capping, and partial or full pulpotomy [1,2,3,4]. Calcium hydroxide (Ca(OH)_2_) is commonly used for direct pulp capping and with sufficient biological responses; however, the main disadvantages of Ca(OH)_2_ are weakness, cohesive strength, marginal leakage, and an inadequate antibacterial effect [5]. MTA is biocompatible with pulp tissue and the surrounding tissue, is a bactericide, promotes pulp healing, and does so without producing inflammation; it does not interfere with the normal processes of root resorption in temporary dentition, and produces a hermetic seal. MTA induces dentinal-bridge formation, and favors the regeneration of periodontal ligament and cementum. It is not resorbable, has low solubility, and a higher radiopacity than dentin [6]. However, traditional MTA formulation has some drawbacks, such as handling difficulties, granular consistency [7], and it is expensive when compared with calcium hydroxide (CH) products in terms of the initial cost of treatment and materials [8]. For this last reason, there is an ongoing quest to find a new and more reasonable pulp-capping agent in terms of cost, setting time, and handling characteristics.

Melatonin (N-acetyl-5-methoxy-tryptamine) is a pleiotropic hormone synthesized in the pineal gland that has important chronobiotic properties [9]. Melatonin acts via high-affinity G-protein-coupled membrane receptors. To date, three different receptor subtypes have been identified in mammals: MT1 (Mel 1a) and MT2 (Mel 1b), and a putative binding site called MT3. Its action through nuclear receptors belonging to the RZR/ROR family has also been described [10]. For these actions, the nuclear receptors of melatonin have been identified in peripheral organs and cells of the central nervous system [11]. 

Some studies suggested that melatonin may play an essential role in the regulation of bone growth, promoting osteoblast differentiation, and stimulating mineralized matrix formation [12,13,14]. Melatonin not only promotes bone regeneration, but also seems to prevent bone resorption through various mechanisms that include osteoblast differentiation and an increase in osteoblastic activity, as well as a reduction in osteoclast differentiation and activity, together with an increase in osteoprotegerin expression and the neutralization of free radicals responsible for bone resorption [15,16,17]. 

Melatonin may play a role in protecting the oral cavity and its use in oral diseases, such as implant placement or periodontitis, was studied by several groups, most of them with favorable results [18,19]. However, little is known about the effects of melatonin on the growth and development of teeth. Emerging evidence suggests that clock genes, a family of genes that control circadian functions within our bodies, also regulate enamel and dentin formation [20,21,22]. A study of human and rat teeth also suggested that melatonin could influence tooth development in a similar way to its action on bone formation. Immunohistochemical analysis revealed that the Mel1a receptor is expressed in secretory ameloblasts, the cells of the stratum intermedium and stellate reticulum, cells of the external dental epithelium, odontoblasts, and in cells of the dental follicle, influencing the regulation of odontoblast-cell function [23].

Melatonin can reduce oxidative-stress levels [24]. Oxidative stress is defined as a disorder in the balance between pro-oxidant species and antioxidants, in favor of the former, which implies the generation of reactive oxygen species (O_2_, H_2_O_2_, OH^−^), reactive nitroxyl species (peroxynitrite), and degradative products of lipid peroxidation (lipid peroxides, malondialdehyde, isoprostanes), which are used to measure oxidative-stress levels. Various antioxidant agents have been used with the aim of unbalancing the oxidation equilibrium, and increasing the capacity of biological systems to rapidly detoxify oxidative reactive species and prevent or repair the resulting damage [25,26,27]. 

Melatonin mixed with cornstarch has not handled difficulties because it has more plasticity than MTA, although it has regular consistency and is cheaper than MTA. As far as we are aware, the possible effect of this material on vital-pulp tissue has not previously been tested. Although no specific evidence has been found, it can be hypothesized that melatonin’s mechanism of action on teeth and odontoblasts might be the same as its action on osteoblasts in the bone. The proven anti-inflammatory action of melatonin encouraged its employment as a pulp-capping agent in the current study. For these reasons, the present study aimed to histopathologically investigate the response of dental pulp in rat molars to melatonin and MTA. The study also evaluated the influence of melatonin when administered orally on the effects of the two study materials and basal-level oxidative stress. 

## 2. Materials and Methods

### 2.1. Materials

The following materials were used: White Pro-Root MTA® (DentsplayMaillefer, Ballaigues, Switzerland) and melatonin (Sigma-Aldrich, St. Louis, MA, USA). The composition of these materials is provided in Table 1.

#### 2.1.1. Animals and Surgical Procedure

Male Sprague Dawley rats, born and raised in the animal facilities of the University of Murcia (Murcia, Spain), were used following Spanish Ministerio de la Presidencia (Cabinet Office) and European Community guidelines for the use of animal experimentation (Royal Decree 1201/2005, law 32/2007, European Directive 2010/63/UE).

Sixteen rats weighing an average of 230 grams were used in the study. Four direct pulp cappings were performed per rat in the first and second maxillary molars, these being healthy and cariesfree. The rats were first anesthetized with an intramuscular injection with a mixture (at 50%) of Rompun® (2% xylazine hydrochloride; Bayer, Kiel, Germany) and Imalgene® 1000 (ketamine chlorhydrate 100 mg + clorobutanol 5 mg; Merial, Barcelona, Spain). The administered dose was 0.2 mL/100 g weight. Tooth surfaces were cleaned with 0.12% chlorhexidine (PerioAid®, Dentaid, Barcelona, Spain). The molar-pulp chambers were then perforated using a tungsten carbide piriform bur with 0.8 mm diameter (KOMET, Lemgo, Germany) driven by a turbine (KaVo SMART torque LUX S615 L, Biberach an der Riss, Germany) with abundant aqueous cooling to prevent the tooth from overheating. Hemorrhaging was controlled using sterile cotton buds and paper points for no longer than five minutes. The study material was placed over the exposed pulp. After this, a base of zinc oxide–eugenol (IRM®, Dentsplay, Tulsa, OK, USA) was positioned, and the cavity obturated with silver amalgam (Syllanoy, TErnaire 94152 Rungis, France).

#### 2.1.2. Study Groups

— MTA group (Group 1; *n* = 16 teeth): Pro-Root MTA® (DentsplayMaillefer, Ballaigues, Switzerland) was used as direct pulp-capping material following the manufacturer’s instructions. The material was left to act over 30 days.
— Melatonin group (Group 2; *n* = 16 teeth): 5 mg of melatonin (Sigma-Aldrich, St. Louis, MA, USA) and 245 mg of cornstarch (Sigma-Aldrich, St. Louis, MA, USA) were mixed with distilled water to a creamy consistency. The mixture was applied to the exposed pulp and left to act for 30 days.
— MTA + Melatonin taken orally group (Group 3; *n* = 16 teeth). Pro-Root MTA® (DentsplayMaillefer, Ballaigues, Switzerland) was used for direct pulp capping following the manufacturer’s instructions and left to act over 30 days. The rats were stored in individual cages and supplied with drinking water containing melatonin (Sigma-Aldrich, St. Louis, MA, USA) in a concentration of 10 mg/100 mL of water that the rats drank ad libitum during the 30 day study period. The average water consumption per animal and day in this group was 38 ± 5 mL.
— Melatonin + Melatonin taken orally group (Group 4; *n* = 16 teeth): 5 mg of melatonin (Sigma-Aldrich, St. Louis, MA, USA) and 245 mg of cornstarch (Sigma-Aldrich, St. Louis, MA, USA) were mixed with distilled water to a creamy consistency. The mixture was applied to the exposed pulp and left to act for 30 days. The rats were stored in individual cages and supplied with drinking water containing melatonin (Sigma-Aldrich, St. Louis, MA, USA) in a concentration of 10 mg/100 mL of water that the rats drank ad libitum during the 30 day study period. Average water consumption per animal and day in this group was 40 ± 4 mL.

The sample size (*n* = 16) was calculated accepting an alpha risk of 0.05 and beta risk of 0.2 in a two-sided test, to find a proportion difference as statistically significant, expected to be 100% in Group 1 and 65% in Group 2. We anticipated a drop-out rate of 10% (sample size and power calculator, https://www.imim.cat/ofertadeserveis/software-public/granmo/).

After the thirty-day study period, the rats were anesthetized with an intramuscular injection of Rompun® and Imalgene®, and blood samples were obtained from the abdominal aorta through laparotomy. When the rats had died from exsanguination, the liver and the kidneys were immediately extracted and frozen at −80 °C for later analysis. The maxillary fragments containing the study molars were separated. 

#### 2.1.3. Optical Microscopy

The maxillary fragments were washed and cleaned of any remaining organic matter, placed in 10% formaldehyde for 15 days in order to fix the tissue, and then for 30 days in TBD-2 (26% formic acid + 8.5% sodium citrate, Anatomical Pathology International, Runcorn, UK) to decalcify the hard tissue. Specimens were dehydrated, processed, and paraffin-embedded. Longitudinal and serial sections (8 µm thick) were then obtained from the samples. According to the methodology described in Fuks et al. [28], every five sections (40 µm) were stained with hematoxylin–eosin (H&E) before optical-microscope observation (Leica DM 5000 B, Leica Microsystems, Wetzlar, Germany). A total of 4 levels were analyzed per specimen.

#### 2.1.4. Histological Evaluation

In order to evaluate the dental-pulp response in all groups, five parameters were evaluated: the degree of pulp inflammation, the presence or absence of pulp necrosis, the presence of a dentinal bridge and reparative dentin across the pulp chambers, the presence and regularity of an odontoblastic layer, and the presence of fibrosis in the pulp, according to the previously recommended evaluation criteria (Table 2) [28,29]. The final score was assessed according to the overall analyzed features. Results were expressed as the percentage of teeth in which the histopathological feature was observed.

#### 2.1.5. Oxidative-Stress Analysis

Thiobarbituric acid reactive substances (TBARS) were determined in plasma, kidney, and liver tissue as a measure of lipid peroxidation by a colorimetric method, as follows [30,31]: 200 µL of plasma sample, or kidney or liver lysate, was used. Then, 0.5 mL of phosphate buffer was added to the samples or lysates. After mixing, 1 mL of a reagent solution was added containing 66 mg% deferoxaminemesylate, 7.5% TCA, 0.25M HCl, and 0.37% thiobarbituric acid. The mixture was vortex-mixed, covered with aluminum foil, and heated at 100 °C for 15 minutes in a heat block (Heatblock II, VWR, Radnor, PA, USA). Once the temperature had dropped to room temperature, TBARS from standards (prepared from 1,1,3,3-tetra ethoxy propane) and samples were extracted into 1 mL 1-butanol. Finally, following vigorous vortex-mixing and brief centrifugation (2500 rpm for 5 min), the color of the butanol layer was read at 532 nm in a spectrophotometer (Biophotometer plus, Eppendorf, Germany). Values are expressed as nmol/mL in plasma samples, and nmol/mg of protein in the kidney or liver tissue ± standard deviation (SD). Protein concentration was measured using a bicinchoninic acid assay kit (Novagen®, Calbiochem, Merck Millipore, Darmstadt, Germany).

### 2.2. Statistical Analysis

Descriptive statistical analysis of the histological data was performed, finding the frequency distribution for each variable. Groups were compared using contingency-table analysis using Pearson’s chi-squared test. This analysis was complemented by residual analysis to determine any significant associations. One-way variance analysis was applied to the plasma, kidney, and liver TBARS assay data.

## 3. Results

### 3.1. Histopathology

**Figure 1:**
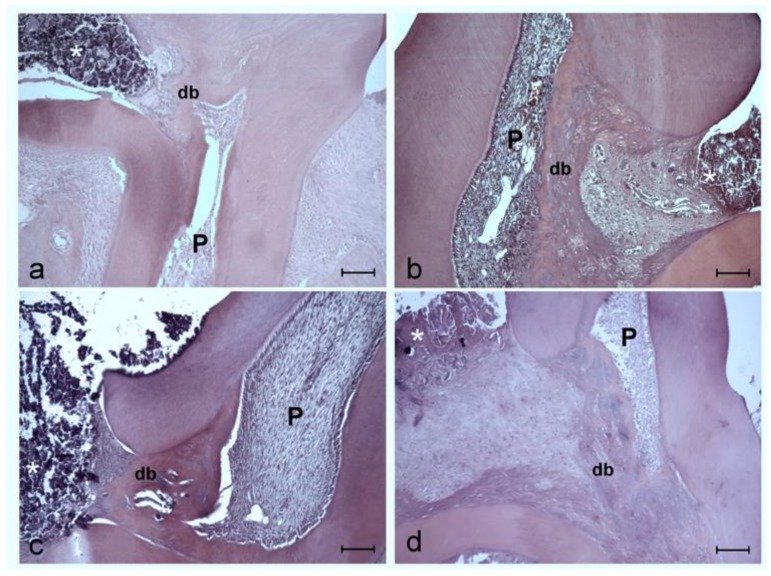
Representative images of area at exposure sites of four study groups at 30 days postintervention. (**a**) MTA group. (**b**) Melatonin group. (**c**) MTA + Melatonin p.o group. (**d**) Melatonin + Melatonin p.o group. All groups showed a well-developed reparative dentinal bridge (db) formed at the exposure interface in contact with the material (asterisk) used for direct pulp capping, with viable pulp (P) and without signs of necrosis. Hematoxylin and eosin stain. Scale bar: 100 micrometers.

Histopathologic analysis revealed that all groups showed a dentinal reparative bridge by day 30 postprocedure (Figure 1), with viable pulp and a regular odontoblastic layer without signs of necrosis. The spontaneous hemorrhagic focus was also identified.

Regarding score analysis, no statistically significant differences were observed concerning degrees of inflammation between the four groups (*p* = 0.108), nor were significant differences found in the degree of necrosis between the treatment groups (*p* = 0.108). For the dentin bridge and reparative dentin variable, no significant differences were identified (*p* = 0.093). Lastly, no significant differences were observed about the odontoblastic layer (*p* = 0.062) or fibrosis (*p* = 0.128) (Table 3).

### 3.2. Oxidative Stress

**Figure 2:**
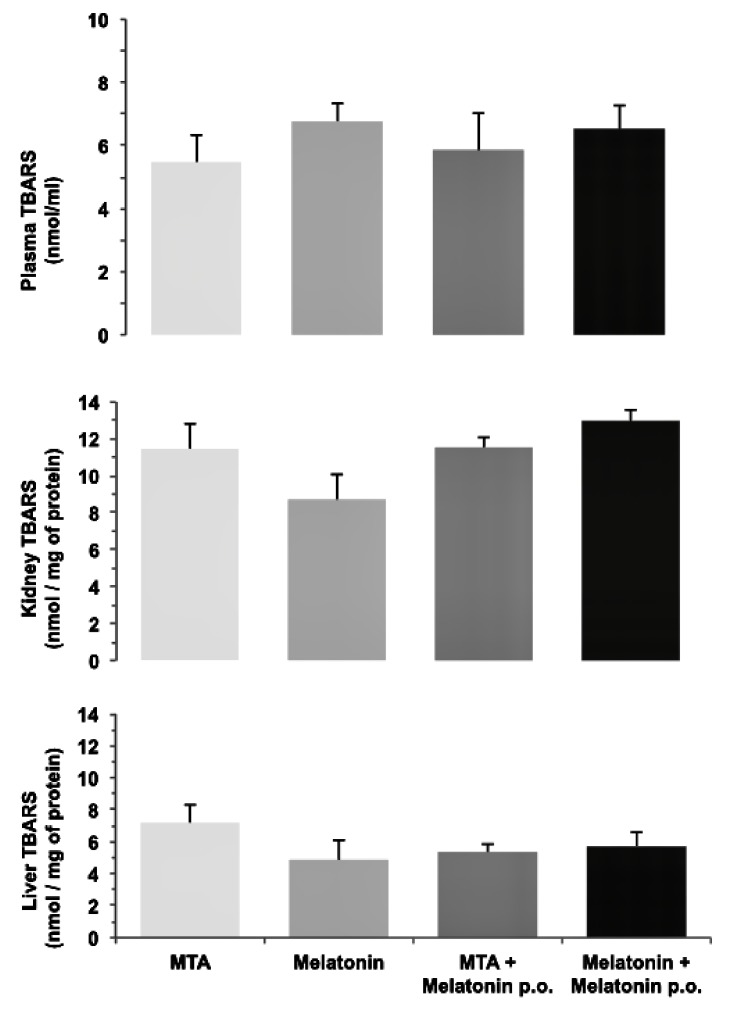
Thiobarbituric acid reactive substances (TBARS) in plasma, kidney, and liver. There were no statistically significant differences between groups.

Differences in oxidative stress, evaluated as TBARS between the four study groups, were not significant, nor were differences in values for plasma (*p* = 0.799), kidney (*p* = 0.130), or liver (*p* = 0.724) (Figure 2).

## 4. Discussion

This is a preliminary study, the first investigating changes in pulp tissue after pulp exposure and direct pulp capping in rats using melatonin. Research performed on rat molar teeth is reproducible in humans [32]. The nonbacteria-associated (without caries) dental-pulp exposure used in the present study excludes any cause for failure of the pulp cap. 

Ideally, the agent used in contact with dental pulp should be innocuous for the tissue and the surrounding structures. MTA is the gold-standard material for dental-pulp capping, and a favorable response was expected when this material was used. Therefore, MTA was used as a positive control material in this study [5].

In the present study, we observed the sequence of reparative processes resulting from MTA application for direct pulp capping after perforation of the pulp chamber, after an inflammatory process that must have happened over the first 15 days following perforation of the pulp-chamber roof [33]. MTA stimulates reparative dentinogenesis and causes localized necrosis that stimulates the secretion of extracellular matrix from odontoblast-like cells [34], and then its calcification to form tertiary dentin [35]. When MTA is applied to pulp cell cultures, it liberates calcium ions to produce osteopontin, osteonectin, and bone-morphogenetic-protein (BMP) expression. Furthermore, MTA can solubilize bioactive molecules, such as the TGF-b growth factor, for new tissue formation [36]. When pulp cells come into contact with MTA, these cells increase vascular-endothelium-growth-factor (VEGF) levels, which is a potent angiogenesis inductor, essential for the regulation of dentin-pulp repair [37].

Results obtained for groups treated with melatonin are similar to those of the MTA group, with 81.25% presenting fibrosis, and 75% forming a dentinal bridge. Histological images showed normal dental-pulp reparative responses: secretion of extracellular matrix by the odontoblasts and odontoblast-like cells, and matrix mineralization with tertiary dentin formation (Figure 1a,b).

In groups where melatonin was used, pulp repair was observed. Although this process has not been described in the literature, it may be hypothesized that melatonin could have a mechanism of action similar to its action on bones. Melatonin provokes stimulating effects on human osteoblast differentiation and activity, and increases alkaline phosphatase activity [38]. Furthermore, in cultured human osteoblasts, melatonin promotes collagen type I expression; it stimulates the production and activity of alkaline phosphatase, osteopontin, bone sialoprotein, and osteocalcin, and the formation of a mineralized bone matrix [39]. Melatonin stimulates osteoblastic differentiation through BMP-2 and -4, and growth factors [40], and reduces the osteoblasts’ differentiation period from 21 to 12 days; these actions take place through membrane receptors [41]. 

Similar effects to these are involved in reparative dentinogenesis promoted by MTA [42], and could be responsible for the effects of melatonin used for the direct pulp capping observed in the present study. Ameloblasts, cells secreting stratum intermedium and stellate reticulum, the cells of the external dental epithelium, osteoblasts, and cells of the dental follicle in humans and rats all express the M1a melatonin receptor during tooth development. In this way, it could be that melatonin regulates the proliferation, differentiation, and function of odontogenic cells, as well as harmonizing tooth growth with the growth of the mandibular bone surrounding it. The M1a receptor, which is the most potent transmembrane receptor for melatonin, is responsible for the bone formation stimulated by melatonin [23]. We could suppose that melatonin would directly exert its action acting on this type of receptor. However, this has to be tested by in vitro experiments.

In the present study, no molar pulp presented inflammation in the MTA groups. This finding could be because the histological study was performed 30 days after perforation of the dentin-pulp complex and direct capping; and inflammatory processes appeared in the first moments, and then gradually disappeared as the pulp underwent adaptive changes. The disappearance of inflammatory phenomena has also been observed in direct pulp capping two months after treatment with MTA [43]. The two molar groups treated with melatonin showed an absence of inflammation (100% and 87.5%, respectively). Reiter et al. [44] affirmed that melatonin is a potent anti-inflammatory agent in numerous experiment models, as it directly neutralizes a wide variety of reactive oxygen species (ROS), including OH_−_, the lipid peroxyl radical (ROO_−_), H_2_O_2_, and singlet oxygen (O_2_^−^). In this way, it regulates NO production through its interaction with the enzymes that synthesize it and, indirectly, the reduction of proinflammatory cytokines and adhesion molecules, which contribute to inflammation and cell damage [44].

As a consequence of reparative actions, the present study observed healthy vital and structured pulp, both in molars treated with MTA (Groups 1 and 3, 100% and 100%, respectively; Figure 1a,c) and in those treated with melatonin (Groups 2 and 4, 100%, 87.5%, respectively; Figure 1b,d), This may be because pulp exposure was technical, not associated with caries, together with a reasonable coronal seal, virtually excluding bacteria as a cause of successful pulp protection. 

Since its discovery in the 1990s, most scientific evidence indicates that MTA is an almost ideal material, and the excellent results it produces do suggest that its use is preferable to other pulp-capping agents [2]. However, its high price has led researchers to seek more economical alternatives that produce similar effects on the reparative processes of the dentin–pulp complex.

The present study set out to determine whether the oral administration of melatonin would have any additional effect on dental-pulp repair, because melatonin is a powerful antioxidant [45]. The results did not identify significant differences between the groups for any of the studied variables (dentin bridge, inflammation, presence of necrosis, presence of odontoblastic layer, and degree of fibrosis). In this way, while the local action of melatonin on dental pulp had similar effects to those of MTA, suggesting that it may be an alternative drug for performing direct pulp capping, its systemic administration did not have any additional benefit. In fact, oxidative-stress values, measured as TBARS in the blood, kidneys, and plasma, were similar with or without melatonin in the water drunk by the study animals after the 30 day study period. It is possible that, as the damage was localized, there was no systemic repercussion in the long term and, therefore, no evident antioxidant effect. Other researchers showed that the exogenous administration of antioxidants such as melatonin [46,47] in healthy animals does not reduce basal-oxidative-stress levels. Nevertheless, antioxidants administered exogenously would accumulate in different tissue types and, following oxidant injury, would prevent the balance from inclining towards oxidative stress in the damaged tissue [48], suggesting that antioxidants may exercise protective effects on the target organs without significantly affecting systemic oxidative stress [30,31].

Lastly, it is possible that the present study’s method was not sufficiently sensitive, and that other oxidative-stress-derived substances rather than lipid-peroxidation products (measured by the present assay) might be implicated in direct pulp-capping-induced injury and consequently in the effects of melatonin, reported as a broad-spectrum antioxidant [49]. Nevertheless, this is unlikely, given that the oral administration of melatonin did not improve the histological parameters compared to the untreated groups.

## 5. Conclusions

According to the results of the present study, melatonin could be used as medication for direct pulp capping, given that its effects were not seen to significantly differ from those of MTA. Melatonin administered orally did not modify the direct-pulp-capping effects produced by either MTA or melatonin. Finally, oral consumption of melatonin by healthy animals did not modify basal level oxidative stress in the plasma, kidneys, or liver.

## Figures and Tables

**Table 1:** Material composition. Note: MTA, Mineral Trioxide Aggregate.

Material	Composition
Pro-Root MTA	Calcium oxide, silicone dioxide, aluminum oxide, bismuth oxide, purified water.
Melatonin	Melatonin 5 mg, corn starch 245 mg, distilled water.

**Table 2:** Scores attributed for levels of evaluated histological criteria [24,25].

Histological Parameters	Scores
Inflammation	0—absence of inflammation
	1—mild inflammation
	2—moderate inflammation
	3—severe inflammation
	4—abscess
Necrosis	0—absence
	1—presence
Dentinal bridge formation and reparative dentin	0—presence
	1—absence
Odontoblastic layer	0—regular
	1—irregular
	2—absence
Fibrotic tissue	0—absence
	1—presence

**Table 3:** Histological results of each experiment group.

Criteria	Degree	MTA	Melatonin	MTA + Melatonin p.o.	Melatonin + Melatonin p.o.
Inflammation	0	100%	100%	100%	87.5%
	1				
	2				12.5%
	3				
	4				
Necrosis	0	100%	100%	100%	87.5%
	1				12.5%
Dentinal-bridge formation and reparative dentin	0	87.5%	75%	81.25%	50%
	1	12.5%	25%	18.75%	50%
Odontoblastic layer	0	100%	81.25%	87.5%	87.5%
	1			12.5%	
	2		18.75%		12.5%
Fibrotic tissue	0		18.75%		12.5%
	1	100%	81.25%	100%	87.5%

No statistically significant differences between groups.
